# Investigating structural and electronic properties of neutral zinc clusters: a *G*_0_*W*_0_ and *G*_0_*W*_0_Г_0_^(1)^ benchmark

**DOI:** 10.3762/bjnano.15.28

**Published:** 2024-03-15

**Authors:** Sunila Bakhsh, Muhammad Khalid, Sameen Aslam, Muhammad Sohail, Muhammad Aamir Iqbal, Mujtaba Ikram, Kareem Morsy

**Affiliations:** 1 Department of Physics, Balochistan University of Information Technology Engineering and Management Sciences, Quetta 87300, Pakistan; 2 Department of Physics, University of Balochistan, Quetta 87300, Pakistan; 3 School of Materials Science and Engineering, Zhejiang University, Hangzhou 310027, China; 4 Institute of Chemical Engineering & Technology (ICET), University of Punjab, Lahore 54590, Pakistan; 5 Biology Department, College of Science, King Khalid University, Abha 61421, Saudi Arabia

**Keywords:** binding energies, CALYPSO structure prediction, DFT, *G*_0_*W*_0_ studies, zinc clusters, zinc isomers

## Abstract

The structural and electronic properties of zinc clusters (Zn*_n_*) for a size range of *n* = 2–15 are studied using density functional theory. The particle swarm optimization algorithm is employed to search the structure and to determine the ground-state structure of the neutral Zn clusters. The structural motifs are optimized using the density functional theory approach to ensure that the structures are fully relaxed. Results are compared with the literature to validate the accuracy of the prediction method. The binding energy per cluster is obtained and compared with the reported literature to study the stability of these structures. We further assess the electronic properties, including the ionization potential, using the all-electron FHI-aims code employing *G*_0_*W*_0_ calculations, and the *G*_0_*W*_0_*Г*_0_^(1)^ correction for a few smaller clusters, which provides a better estimation of the ionization potential compared to other methods.

## Introduction

Zinc is a group-IIB element that is frequently used as a galvanizing material and in storage media as an anode [1–3]. However, its ability to lose electrons quickly to oxygen makes it unsuitable as a coating material. Zinc exhibits a s^2^ closed-shell structure, and its dimer forms through van der Waal (vdW) forces [4]. As the cluster size increases, the properties of the clusters change significantly, and the effect of vdW forces decreases. Bulk zinc has metallic characteristics because of the overlap of the s and p orbitals. In the past, Zn clusters have been analyzed both experimentally and theoretically, where the studies were mainly conducted to determine the stability and electronic properties of the zinc cluster ground state [4–6]. The majority of the research work on Zn clusters is focused on the vdW transition for the Zn clusters. For example, Wang et al. [7] investigated it by using the PW91 functional, which suggested that the transition starts from *n* = 8. Iokibe et al. [8] obtained a similar result using density functional theory (DFT) calculations at different levels of theory to study the transition states (vdW to semiconductor-like states) in Zn clusters. In addition, the approaches used to study the electronic properties, such as ionization potentials (IPs) of zinc, are based on the ∆-SCF methods; for some clusters, such as Zn_2_, the results significantly underestimate the experimentally measured IPs. State-of-the-art approaches, such as *GW* approximation, have been proven to provide accurate IPs and electron affinity (EA) values for various clusters [9–12].

Determining the ground states of clusters is essential; several metastable isomers are present in an experimental study, which can introduce difficulties in determining the ground state structure. Several algorithms have been used to describe the ground state and low-lying structures of the clusters. Among these approaches, particle swarm optimization (PSO), combined with density functional approximations, was used to determine the ground state structure. Thus, one can efficiently locate the global minimum in the potential energy surface. Based on the PSO algorithm, Wang et al. [13–14] developed a code called CALYPSO (“Crystal Structure Analysis by Particle Swarm Optimization”). It has been used previously by many researchers in discovering new materials [15–16]. In addition to the ground state properties, electronic properties such as ionization energies (IEs) and HOMO–LUMO gaps are also important, as they determine the physical properties of the clusters. Previous attempts to study the electronic properties of Zn clusters based on ∆-SCF methods tended to underestimate the ionization energies of the clusters as the size grew. State-of-the-art techniques, such as the *GW* method, can effectively describe the electronic properties of many clusters with higher accuracy. In addition to *G*_0_*W*_0_, we have also applied the 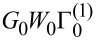 correction. The unavailability of experimental data is a key issue because of which the determination of ground state and electronic properties remains unexplored. To our knowledge, no *G*_0_*W*_0_ studies on neutral zinc clusters have been reported in the literature. Our *G*_0_*W*_0_ calculations will provide a benchmark to help accelerate the research on clusters and creating materials with high stability that can be used for advanced energy storage applications [17–19].

In this work, we have employed the generalized gradient approximation (GGA) to optimize the results and to obtain the ground state structures and the isomers of neutral Zn clusters. Furthermore, we have performed *G*_0_*W*_0_ calculations using the FHI-aims all-electron code to study electronic properties such as IPs, electron affinities (EAs), and HOMO–LUMO gaps of Zn clusters. In addition, the study of low-lying isomers is also carried out to compare the metastable structures with the ground state, which ensures that the obtained lowest-energy structure is the actual ground state.

## Computational Methods

All geometric optimization calculations were carried out with the PBE exchange–correlation functional of the GGA. The structure prediction in our work was carried out by the CALYPSO code [20–21] with ABACUS software for structure optimization [13–14]. The non-relativistic ONCV-type pseudopotential (SG15 V1.0) [22] was used. The obtained structures have been carefully analyzed with the VESTA software, and low-energy isomers were refined from more than 600 structures (ca. 22 generations in CALYPSO).

The geometric optimization of all clusters for a size range of *n* = 2–15 was performed in two steps: (i) structure search and initial geometric optimization, within which the distinct structures were separated using the GGA (PBE) functional, and (ii) high-precision optimization by ABACUS using the “accurate” setting. PBE instead of the hybrid functional was used because of its low cost; also, it brings a non-empirical functional that can easily and reliably predict new systems and properties. The next step was to perform the *G*_0_*W*_0_ calculations using the FHI-aims all-electron package [23–24], which was further used to assess the electronic properties of the Zn clusters. We used the pre-relaxed structures to obtain the energy gaps and IPs of the Zn clusters. In FHI-aims, *G*_0_*W*_0_ calculations employing the NAO basis sets were performed with the PBE functional to relax the structure with “tier 4” and “tight” settings. The results obtained from *G*_0_*W*_0_ calculations predict better IPs and energy gaps of the molecules and clusters. The results are compared with the previously available experimental and theoretical data to validate our work.

## Results and Discussion

In this section, we will present the results of the structural relaxation, stability, and electronic properties of neutral Zn clusters. The predictions of the various geometrical structures are presented, and binding energies are discussed. In addition, we have also explored the ionization potentials, electron affinities, and energy gaps for the series of Zn clusters.

### Geometrical structures

**Figure 1:**
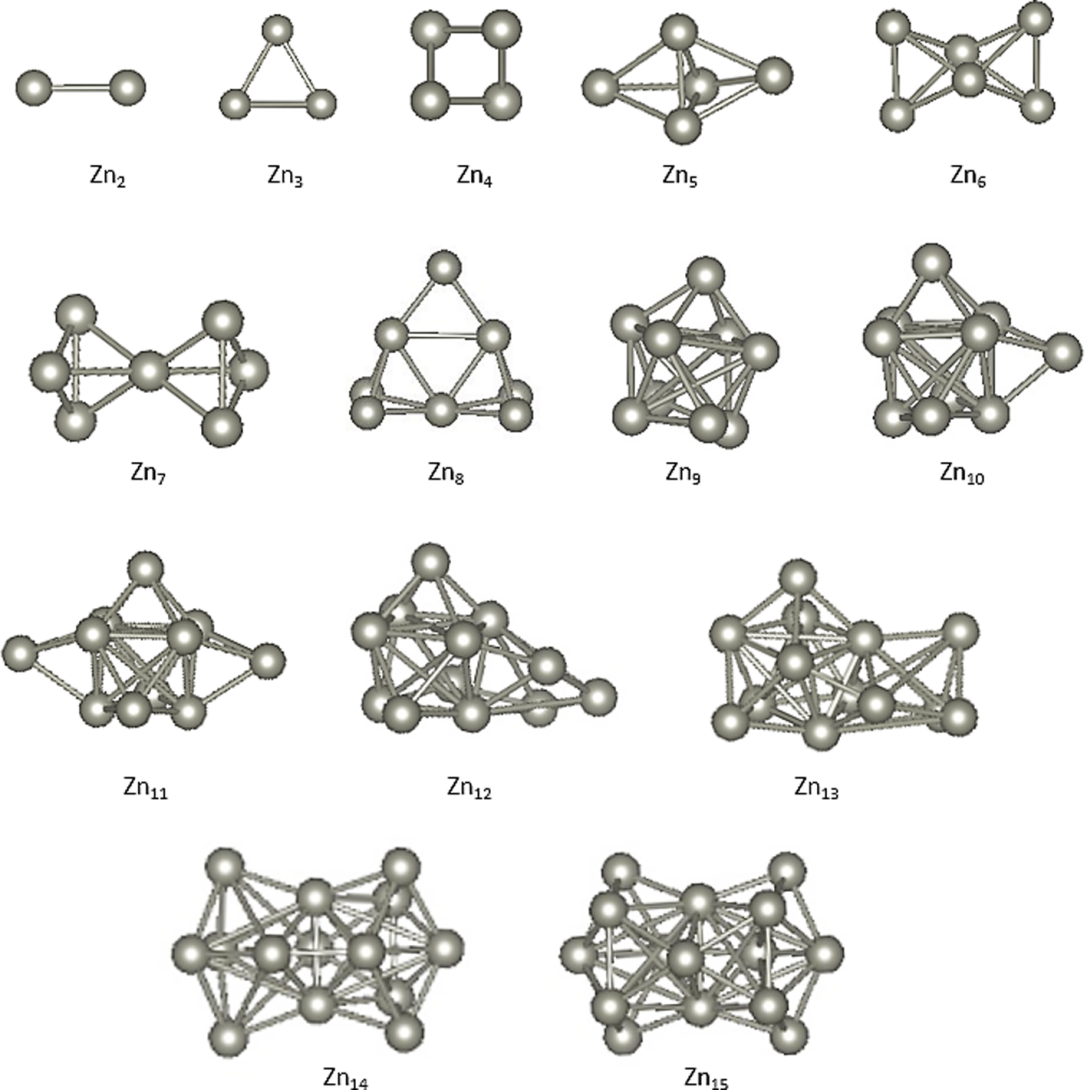
Geometrical structures of ground state Zn clusters.

Various theoretical studies have been performed regarding the structural properties of Zn clusters. Among such, a DFT study employing the PBE functional revealed that the symmetric structures are less stable than the structures with lower symmetry [6]. There are also experimental and theoretical studies to determine the binding energies of Zn clusters [4–5,25]. In addition, in an experimental study also validated with a DFT approach, Aguado et al. calculated the binding energies of neutral and ionic Zn clusters [26]. The ground state structures of neutral zinc clusters obtained in our work are shown in Figure 1. The ground state structures for sizes of *n* = 3–7 follow previous reports [6–7,27–28]. The trimer and pentamer structures have *D*_3_*_h_* symmetry, whereas the tetramer belongs to a *T_d_* point group.

The Zn clusters show planar geometrical structures for *n* = 3–4. The geometrical structure of Zn_8_, a magic cluster obtained in this work, is similar to that of Chaves and co-workers [29]. The octamer of zinc is particularly interesting, as the transition from vdW forces to metallic bonds occurs at *n* = 8. However, the geometrical structure of the ground state is still controversial. Recently, Chaves et al. [29] studied various transition element clusters and found a new ground state of the zinc cluster with *n* = 8. The zinc cluster with nine atoms was also obtained by Iokibe et al. [6], who predicted the Zn_10_ structure obtained in our work. They used the PW91 functional to calculate the binding energies of medium-sized Zn clusters. The cluster geometries from *n* = 11–15 were also predicted by Li et al. using the B88 functional. The structure of Zn_14_ was also obtained as the second lowest in our study and by Wang et al. [7]. This structure was reported as the lowest-energy structure.

### Stability and binding energies

In order to study the stability of the generated structures, it is essential to determine the binding energy per atom of each cluster isomer, which can be defined as:


[1] $$\frac{E_{\text{b}}}{n}=E_{\text{atom}}-\frac{E_{\text{tot}}}{n},$$

where *E*_tot_ is the total energy of the cluster after relaxation, *n* is the size of the cluster, and *E*_atom_ is the energy of a free atom. Here, we have also employed spin-polarized calculations to obtain the binding energies of Zn clusters. For metallic systems, spin effects significantly influence the total energies, and neglecting these effects can result in overestimating binding energies. The dimer binding energy obtained in our work is 0.022 eV, close to the experimental value of 0.03 eV [30]. We have also calculated the binding energy of the zinc dimer by using the FHI-aims code and PBE relaxed calculations. The output geometries from CALYPSO and ABACUS relaxed calculations are used as input, and the PBE relaxed calculation is performed in the FHI-aims code. The obtained binding energy from the FHI-aims code for the dimer is 0.034 eV, the same as the experimental binding energy. It should be noted that the lower binding energy of zinc dimers is mainly due to the weak vdW bonding effects [6,8].

**Figure 2:**
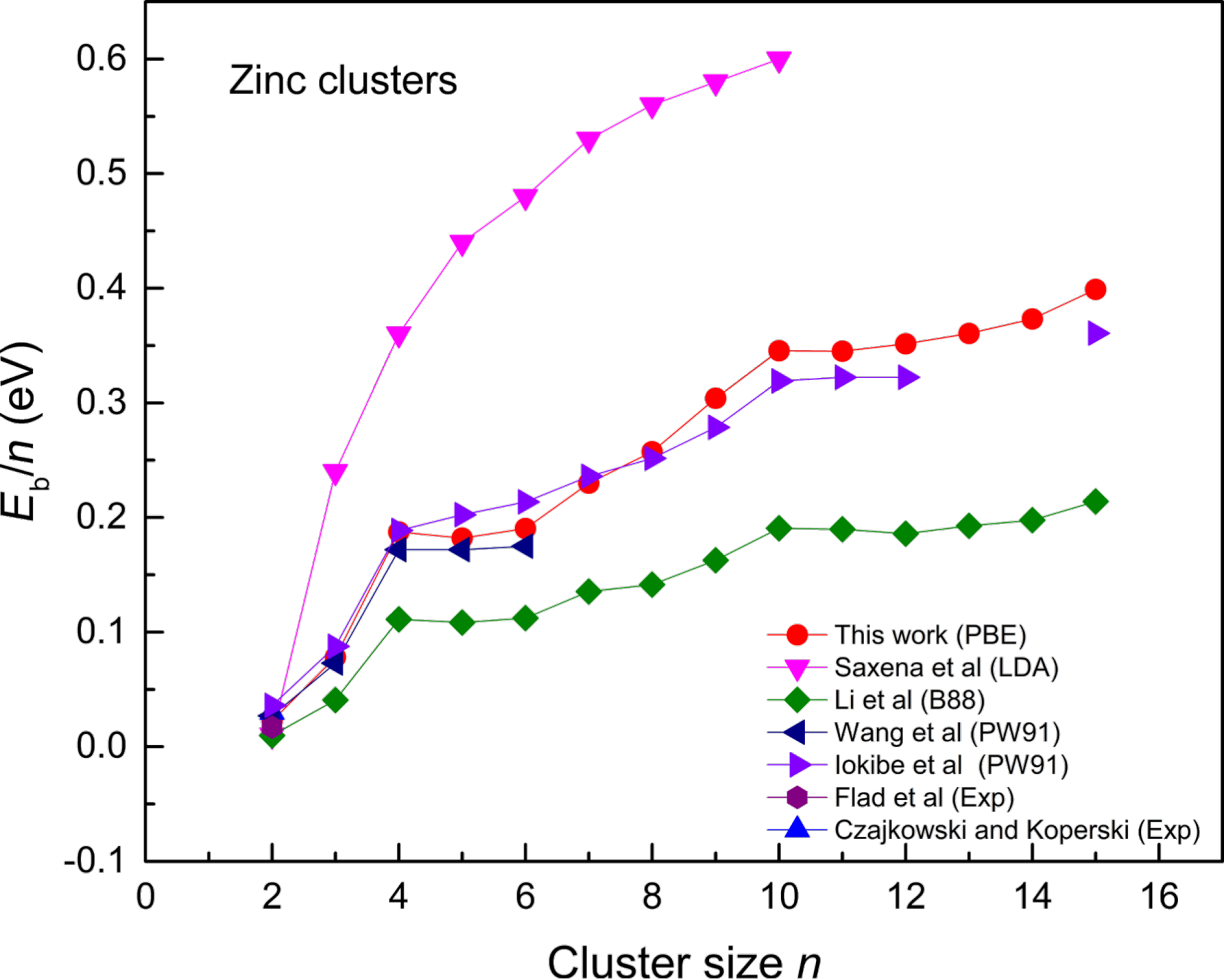
Binding energy per atom (eV) of zinc cluster ground states for the size range *n* = 2–15 compared with experimental [30–31] and theoretical works [1,6,27,32].

Figure 2 shows that the binding energies increase swiftly up to a cluster size of *n* = 4. For further increase in the cluster size, the curve slightly flattens (up to *n* = 6). There is a sharp increase in the curve for *n* = 7, 9, and 10. These high binding energies display the higher stability of these particular clusters, which is consistent with the shell model and spectroscopic observations. Wang et al. [7] also reported these peaks in theory for clusters of *n* =4, 7, 9, 10, and 14. It can also be seen in Figure 2, that there are two shoulders or peaks, one at *n* = 4 and the other at *n* = 10.

As zinc has two valence electrons, these peaks correspond to the formation of magic number clusters. Eight and 20 valence electrons make a cluster more stable, which is seen as a peak in the binding energy curve of Zn clusters. For cluster sizes of *n* = 11–15, *E*_b_ increases smoothly. Our predicted binding energies are similar to those from an earlier study by Iokibe et al. and Wang et al. [6–7], who determined the stability and structural properties of neutral Zn clusters by employing the PW91 functional. Analyzing the performance of different functionals for Zn clusters, it can be concluded that the LDA overestimates the binding energy significantly. Our PBE results are in good agreement with the PW91 results reported by Iokibe et al. [6] and Wang and co-workers [7].

### Electronic properties

**Figure 3:**
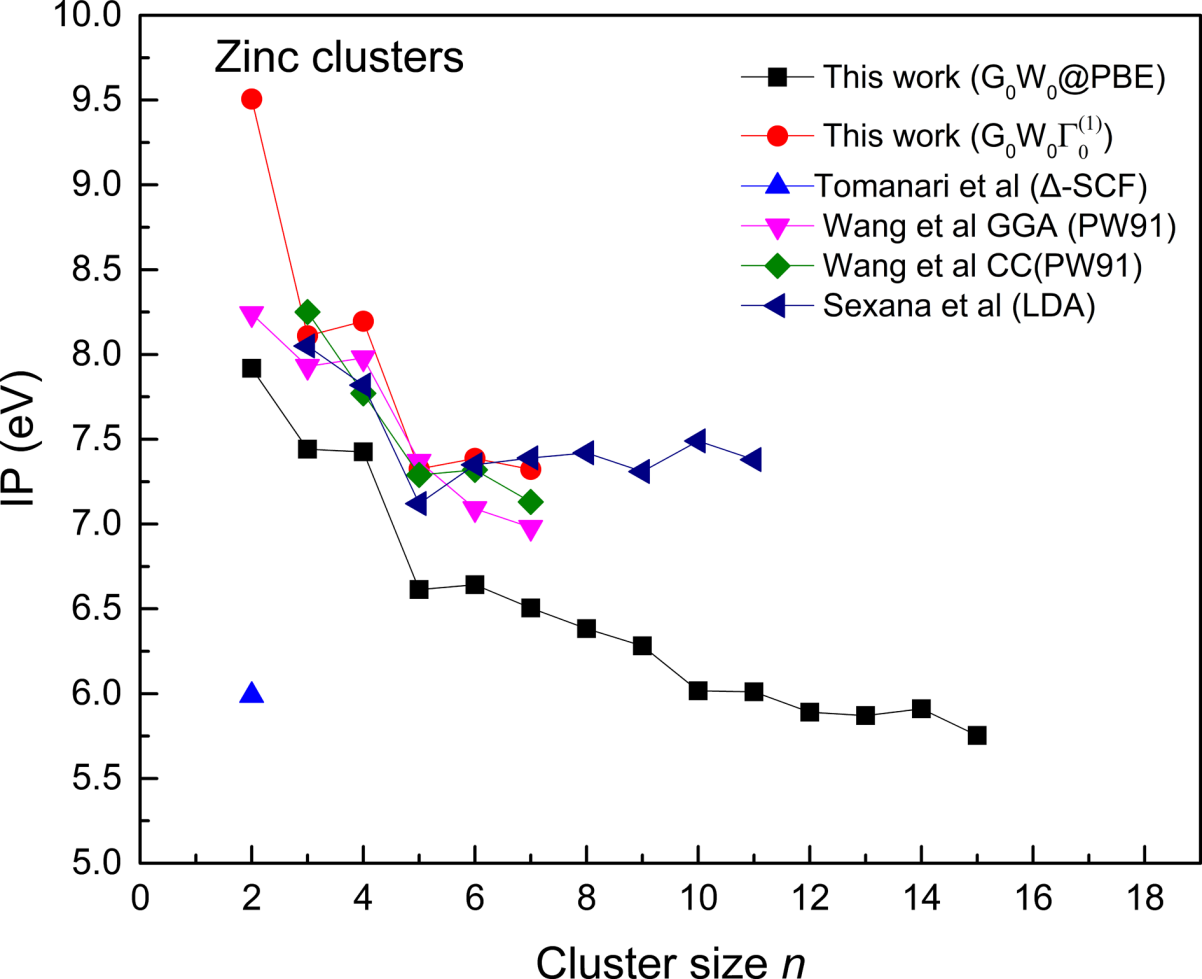
Ionization potentials of Zn clusters for the size range *n* = 2–15. Comparison of our work *G*_0_*W*_0_@PBE with PW91 [7], ∆SCF [33], and LDA [32].

The ionization potential obtained from *G*_0_*W*_0_ calculations for the size range of *n* = 2–15 is plotted and compared with literature in Figure 3.

In the *G*_0_*W*_0_ scheme, the IPs are obtained from the negative of the quasi-particle HOMO level. The curve shows a steep drop in IPs for the cluster sizes *n* = 4–5, as predicted earlier [7]. Moreover, high IPs are obtained for clusters *n* = 4, 6, 9, 11, and 14 in our work, which shows that these structures are relatively more stable than the other clusters. It is also interesting to see that the typical even–odd oscillations are absent for zinc clusters; however, the prominent peak showing the higher IP on the curve is obtained at *n* = 4. The *G*_0_*W*_0_ calculations yield significantly lower IPs compared to the other schemes such as LDA and GGA. The dimer IP from the ∆-SCF scheme is fairly low compared to the other schemes. In addition to the *G*_0_*W*_0_ calculations, we have also applied the 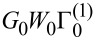 correction to the smaller clusters (Figure 3). The IPs obtained from the 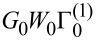 correction show a good estimation compared with reported literature and our *G*_0_*W*_0_ calculations. For the dimer, the IP obtained from the 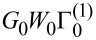 correction is closer to the experimental value of 9.0 ± 0.2 eV. IPs from a model Hamiltonian presented by Tarento [34] suggested a value of 8.61 eV, and our IP is the closest to the reported experimental result. Furthermore, the 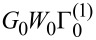 correction for a cluster size of *n* = 2–7 was used to compare our IPs from *G*_0_*W*_0_ calculations. The 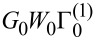 correction has also shown similar trends in IPs as in the literature. The 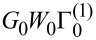 schemes turned out to yield better results than other schemes as seen in Figure 3. Except for the dimer ionization potential, which is higher than those from the other functionals, the 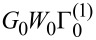 correction yields a good approximation for the IPs. As there is no experimental work so far, one needs to rely only on reported theoretical works. Our 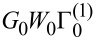 benchmark is closer to the predicted theoretical results. In addition to the ionization potential study, we have also obtained results for the electron affinity from the *G*_0_*W*_0_ method. The electron affinity is taken as the negative of the LUMO level. The results are presented along with energy gap (*E*_gap_) and hardness in Table 1.

**Table 1:** Electron affinity, *E*_gap_, and hardness for neutral clusters of zinc.

Cluster size *n*	This work *G*_0_*W*_0_@PBE EA (eV)	This work *E*_gap_ = IP − EA (eV)	*E*_gap_ PW91^a^ (eV)	*E*_gap_ LDA^b^ (eV)	Hardness η = (IP − EA)/2 (eV)

2	0.65	7.27	4.505	4.410	3.63
3	0.12	7.56	4.000	3.421	3.78
4	0.41	7.02	3.516	3.556	3.51
5	0.53	5.89	3.053	2.410	2.95
6	0.72	5.92	2.989	2.433	2.96
7	1.43	5.47	2.758	2.230	2.74
8	1.24	5.14	2.568	2.275	2.57
9	2.01	4.27	1.642	1.129	2.14
10	1.43	4.59	1.979	2.006	2.29
11	1.19	4.82	2.126	—	2.41
12	1.69	4.21	1.768	—	2.10
13	1.75	4.12	1.726	—	2.06
14	1.85	4.06	1.726	—	2.03
15	2.16	3.59	1.516	—	1.80

^a^Ref. PW91 [7]; ^b^Ref. LDA [32].

**Figure 4:**
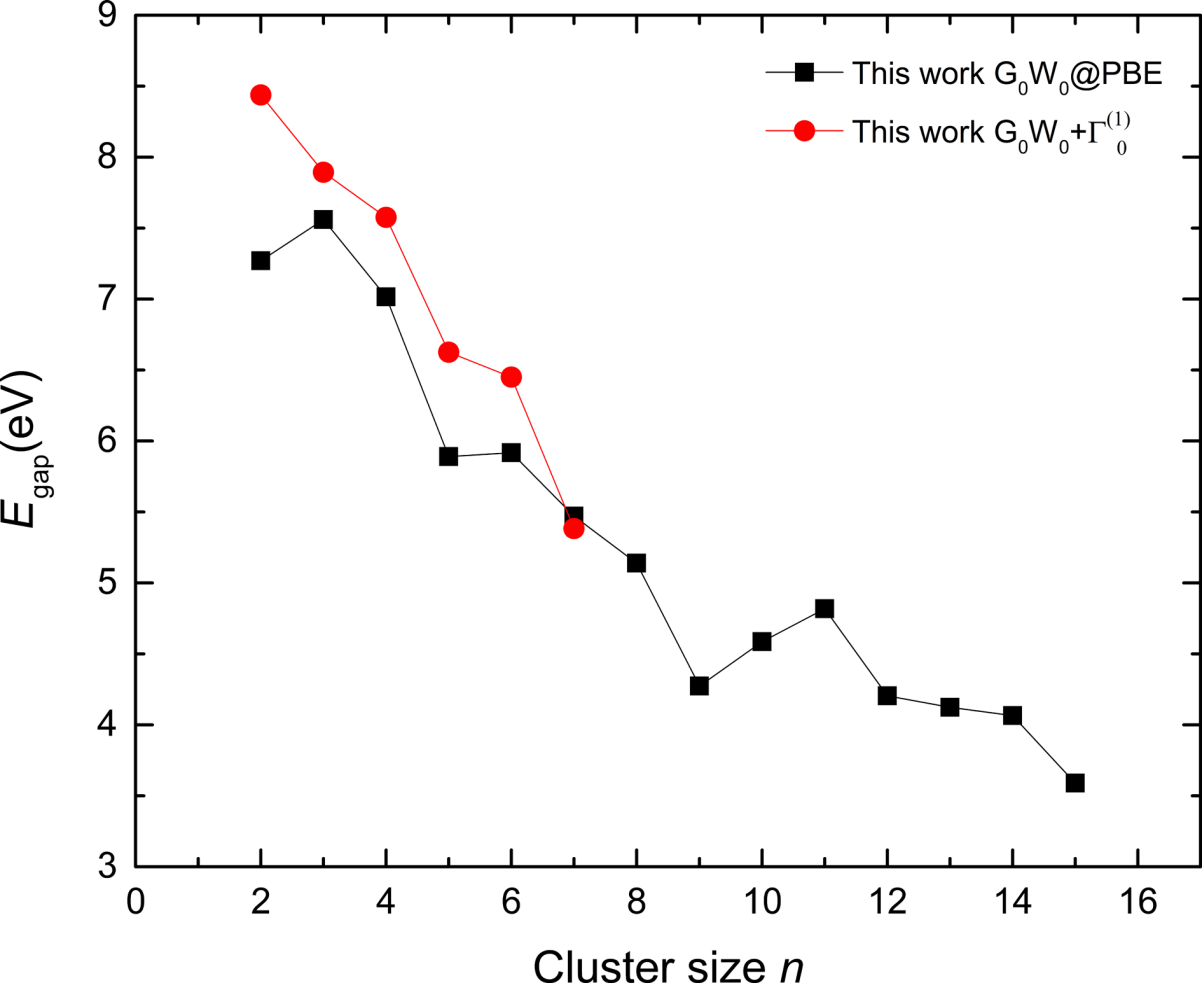
Comparison of *E*_gap_ of neutral Zn clusters (*n* = 2–15) from *G*_0_*W*_0_ and 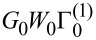 calculations.

Our estimated EAs for Zn clusters are close to those obtained by Dai et al. [28], who also predicted high electron affinities of the cluster Zn_9_. The reported literature shows that IP and *E*_gap_ are also slightly lower than those of the neighboring clusters [7]. The *GW* scheme has also been proven effective for the description of accurate bandgaps. A comparison of the energy gaps is presented in Figure 4, in which the results from reported literature is plotted against our *G*_0_*W*_0_ and 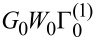 results. It can be seen from the graph that our *G*_0_*W*_0_ and 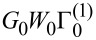 results are in good agreement for cluster sizes of *n* = 3–7. For the cluster size *n* = 7, our estimated bandgaps from 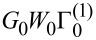 calculations are similar to those obtained from *G*_0_*W*_0_ calculations. The bandgaps follow a decreasing trend, which follows the behavior of metallic bandgaps. One exception is the zinc dimer, for which our bandgap from 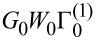 calculations is relatively high, which may be attributed to the van der Waals forces.

The HOMO–LUMO gap trend shows that, at larger sizes, the behavior of the cluster becomes close to that of the bulk material, that is, the HOMO–LUMO gap decreases. As seen in Figure 4, the bandgap values obtained from *GW* calculations are far from the bulk limit of metals. In our work, the HOMO–LUMO gap decreases from ca. 8.5 eV to approximately 3.5 eV, but it is still sizable compared with the bulk metal energy gap. Apart from this situation, for small-size clusters or nanoparticles, it is possible to observe quantum confinement effects resulting in an indeterminate bandgap, such as in the case of semiconductor clusters. As the size of the cluster decreases, the electronic energy levels become quantized, which is useful regarding tuning the bandgap for material engineering.

## Conclusion

The ground state and electronic properties of zinc clusters for sizes *n* = 2–15 are studied in this work. Structures of the Zn*_n_* clusters have been generated using the CALYPSO code (interfaced with ABACUS) using the PBE functional. The binding energies show the two-knee behavior also seen in metal clusters of various species. Electronic properties, such as IP, EA, and the relative hardness of the clusters, have been obtained using the *G*_0_*W*_0_@PBE in FHI-aims. The IP benchmark shows the size evolution behavior of the Zn clusters. The benchmark results are in good agreement with previously reported data. Moreover, the 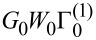 corrections showed substantially improved ionization potential values compared to the *G*_0_*W*_0_ approach.

## Data Availability

The data that support the findings of this study are available upon request from the author (Dr. Sunila Bakhsh).
